# Public health nurses' experiences of assessing disruptive behaviour in children and supporting the use of an Internet‐based parent training programme

**DOI:** 10.1111/scs.12744

**Published:** 2019-09-05

**Authors:** Terja Ristkari, Kaisa Mishina, Milka‐Maija Lehtola, Andre Sourander, Marjo Kurki

**Affiliations:** ^1^ Department of Child Psychiatry University of Turku Turku Finland; ^2^ Turku University Hospital Turku Finland; ^3^ Department of Nursing Science University of Turku Turku Finland; ^4^ INVEST Research Flagship University of Turku Turku Finland

**Keywords:** nursing, public health nursing, primary health care, mental health, parenting, survey, competence

## Abstract

**Background:**

In Finland, although families generally receive support from child health clinics, some need more help in dealing with their child's emotions, behaviour and psychosocial development. Public health nurses play a central role in providing vital psychosocial support for families, but they often lack the confidence and competence to tackle mental health problems.

**Aim:**

To describe how public health nurses used and experienced a working model that combined a psychosocial tool (Strengths and Difficulties Questionnaire, SDQ) to identify disruptive behaviour in four‐year‐old children and an Internet‐based parent training programme with telephone coaching.

**Methods:**

This is a descriptive, cross‐sectional survey study. The sample consists of public health nurses (n = 138) who were working in child health clinics in Finland that had used the working model. Statistical data were analysed using SPSS Statistics for Windows. The responses to an open‐ended question were analysed using inductive content analysis.

**Results:**

The experiences about the working model were mainly positive. The public health nurses felt that the psychosocial tool, the SDQ, was easy and suitable to use in child health clinics. The availability of an Internet‐based parent training programme provided greater support for parents by overcoming practical barriers. Overall, the working model helped nurses to develop their mental health competencies.

**Conclusion:**

Within primary care, the need to tackle psychosocial problems is increasing, and for this, public health nurses need extra support and tools. It seems that the working model, including the SDQ and the online and telephone coaching programme, worked well in child health clinics. This working model can be used to provide parental support and improve nurses' mental health competencies.

## Introduction

For nearly a century, the Finnish maternity and child health clinic system has provided low‐threshold services for families with children, covering the vast majority (99.6%) of the child population 1. The child health clinic system plays a central role in Finnish society and is absolutely vital in preventing diseases and promoting public health. Typically, child health clinics offer 15 to 20 health check‐ups during childhood 2. Of those, three are extended, legally required check‐ups 3 that are conducted when the child is four months, 18 months and four years old. The last extended check‐up particularly focuses on the interaction between the child and parents, as well as the child's social and cognitive skills 2.

Despite the strong support provided by the child health clinics, parents still feel that they need more support with regard to their child's emotional life, behaviour and psychosocial development 4. Many parents express concerns about their child's behaviour, but agreement on these concerns is relatively low with child health professionals. 5. In fact, public health nurses tend to have little knowledge of mental health 6, 7, and they often lack the confidence and competence to confront mental health problems 6, 8. On the other hand, public health nurses' attitudes towards mental health are positive 6, and this provides an excellent opportunity to increase their competence and skills with regard to mental health promotion.

Disruptive behaviour in childhood indicates a wide range of negative outcomes for the child later in life 9, 10, 11, 12. Parent training has been found to be the most effective way to prevent and treat disruptive behaviour in children 13, 14, 15, 16. However, to our knowledge, only a few evidence‐based, low‐threshold and easily accessed parent training interventions are currently available. Therefore, we worked closely with child health clinics to develop a model that provides population‐based identification of children at risk of disruptive behaviour and a parent training intervention. The parent training intervention was an Internet‐based parent training programme that provided 11 weekly themes and associated telephone sessions with a healthcare professional with specifically trained for a parent training programme. The effectiveness of this parent training has been proven, with short‐term 16 and long‐term (24 months) positive effects 17. The rationale behind the programme is that it can be accessed by parents at any time, at home or during work breaks, and it overcomes barriers such as travelling to appointments, work absences and the stigma of having a child with disruptive behaviour. The core content addresses positive parenting skills and problem‐solving abilities using interactive and multimedia components, such as exercises and video clips.

However, although there is strong evidence behind the effectiveness of this intervention 16, 17, the true practical implementations can be challenging. Therefore, the crucial question was how to implement the working model in a way that public health nurses could fully utilise it as part of their daily practice.

After a randomised controlled study confirmed the effectiveness and feasibility of the Internet‐based parent training intervention 16, the implementation study 18 was initiated in 2015 with the aim to implement the working model in child health clinics. This survey study is part of the implementation study. Public health nurses were an essential element of this working model, as they are the gatekeepers for the intervention and partners in implementing the whole working model in primary care. Therefore, they were offered systematic training and a practical, psychosocial identification tool to assist them in their role in promoting children's mental health and psychosocial well‐being. The training included basic knowledge about disruptive behaviour in children and details of the Internet‐based parent training intervention and its content.

**Figure 1:**
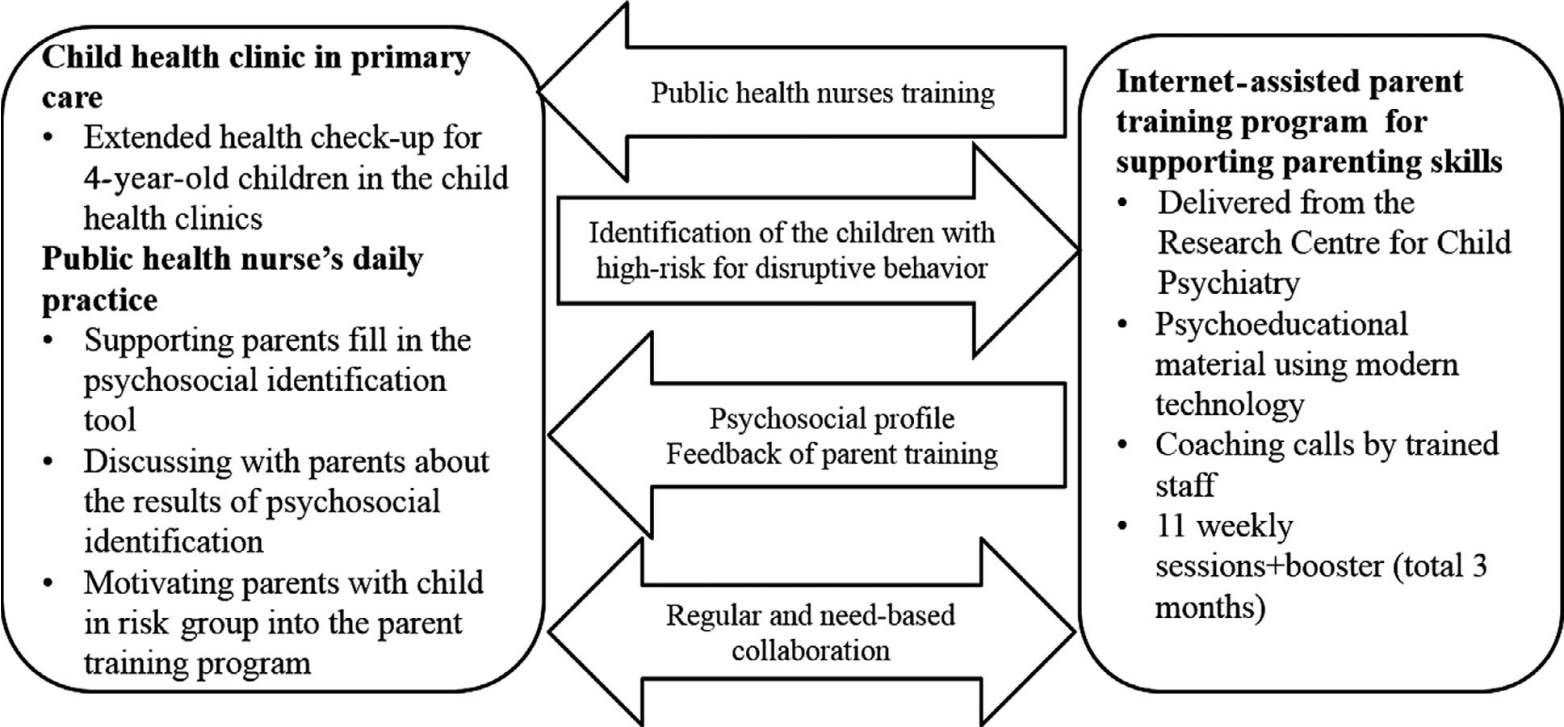
Process of the parent training working model.

The psychosocial identification tool was a Finnish version of the Strengths and Difficulties Questionnaire (SDQ) 19, 20, 21. The tool was used to provide individual‐level results for four‐year‐old children at the time of their last legally required extended health check‐up. This tool enabled public health nurses to identify children who posed a high risk for disruptive behaviour, and they were trained in its use and encouraged to discuss the results with parents and doctors. If a child's scores indicated disruptive behaviour, the Internet‐based parent training intervention was offered to the families by the trained staff from the Research Centre of Child Psychiatry at the University of Turku. The process and roles regarding the working model are outlined in Figure 1.

This study is a part of a project, the flagship Inequalities, Interventions, and New Welfare State (INVEST), which aims to increase the well‐being of Finnish society from childhood to adulthood and prevent psychosocial risks through innovative interventions. The overall goal of the INVEST Research Flagship is to provide an outline for a more efficient, just and affordable model of the Finnish welfare state.

## Aims

**Figure 2:**
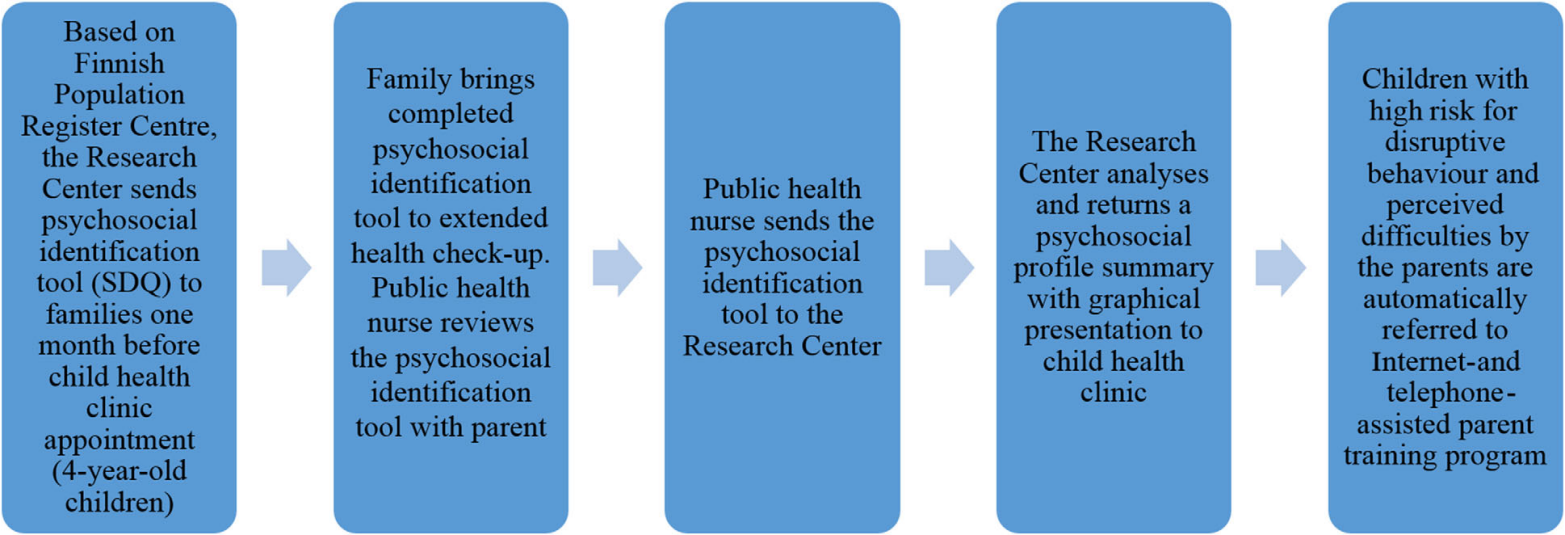
Practical process of the working model using psychosocial identification and parent training in child health clinics.

The aim of this study was to describe the use and experiences of public health nurses concerning the working model implemented in the child health clinics in which they worked. The working model consisted of the psychosocial evaluation tool (SDQ) and a parent training programme provided by the Research Centre of Child Psychiatry. Figure 2 explains how the actual process worked.

## Methods

### Design and sample

The study was a desriptive, cross‐sectional survey consisting in electronic questionnaire.

The study took place in Finland, a Nordic welfare state that provides public health services. This study comprised 95 child health clinics in the cities of Tampere, Espoo, Kouvola, Kuopio and Turku, the towns of Hyvinkää, Naantali and Parainen, the municipalities of Mäntsälä and Tuusula, and two provinces, the Eksote and the Kainuu Social Welfare and Health Care Joint Authority. All of these areas were included in the implementation study and were therefore selected to take part in this survey. This study aims to more deeply understand the implementation plan of the working model when utilising an evidence‐based intervention into practice in a real‐world setting 18. As a part of the implementation plan, we identified implementation drivers which needed to take the implementation process into account. One of the core implementation components was the training provided for the public health nurses who were the key actors in successful implementation 22. A half‐day of training consisted of the theoretical background of the working model and a basis of preventive mental health 18.

All 277 public health nurses working in the child health clinics involved in the implementation study were asked to participate. Of those, 138 (50%) participated and completed the whole survey.

The data were collected between April and May 2017 using an electronic survey that included questions about background information as well as experiences and actual use of the working model. A link to the survey was emailed to all public health nurses included in the implementation study. Participants were given 2 weeks to fill in the questionnaire anonymously before receiving three reminders at two‐week intervals.

### Measures

Background information included questions about the age and gender of the public health nurses. They were also asked about where their child health clinic was based, their professional career and how long they had worked as a public health nurse.

A five‐point Likert scale questionnaire was developed specifically for this study. The questionnaire was based on the Finnish national public health nurses' professional competence areas aiming to systematise the quality of the education and developed by the specialists of 21 universities of applied sciences who were responsible for the education of public health nurses in Finland 23. An expert panel of three researchers having backgrounds in nursing, public health nursing or education examined the questionnaire for content and clarity. The instrument consisted of 11 statements, seven closed questions and one open‐ended question. First, there were 11 statements about how the working model applied to the competence areas, ranging from one (totally agree) to five (totally disagree). Second, there were seven closed questions and one open‐ended question to assess the experiences of the public health nurses regarding how relevant the working model was to their work and how they had used the working model in their daily practice.

### Ethical considerations

This research was part of a larger implementation study and therefore was covered by the ethical approval (25/2018) from the University of Turku gained for the wider study. The principles of research ethics 24 were followed. The researchers respected and protected the participants' autonomy, privacy, dignity and integrity at every stage of the study. The participants were health care professionals having experience with and training for the working model. They were informed about the voluntary nature of the study, and their anonymity was ensured throughout the study. Completion of the questionnaire was taken as consent to participate. The questionnaire included information regarding demographic factors of the public health nurses, but their names and working organisation were not recorded. The questionnaire was sent directly to the researchers, and the participants' organisations were not informed about the individual responses of the respondents.

### Data analysis

The quantitative data from 138 respondents were analysed with descriptive statistics using SPSS Statistics, version 21.0 for Windows 25. The open‐ended questions with 58 respondents supplemented the quantitative data. The open‐ended questions were analysed with inductive content analysis by searching for similarities and differences in the data 26, 27. Firstly, the meaning units were selected, and the text was assigned to one of the units. Secondly, the meaning units were grouped based on similarities and differences, and these groups were named (codes). Thirdly, the codes were divided into sub‐themes and named. Fourthly, the named sub‐themes were compared and grouped based on similarities and differences. This finally formed the themes, which were named as content characteristics.

## Results

### Study participants

The 138 study participants were all females, and most (n = 105, 76%) were more than 36 years of age. Most of them (n = 121, 88%) had more than five years' working experience as a public health nurse, and most had held their current job for more than five years (n = 109, 79%). A more detailed description of the background characteristics is provided in Table 1.

**Table 1:** Background characteristics of the participants

Characteristics	n	%
Age (years)		
<25	4	3
25–35	29	21
36–46	41	30
47–57	42	30
>57	22	16
Gender n (%)		
Females	138	100
Males	0	0
Number of years working as a public health nurse		
<1	2	2
<5	15	11
5–9	29	21
>10	92	67
Number of years in current role		
<1	6	4
<5	23	17
5–9	45	33
>10	64	46

### Benefits of the working model in the clinical practice of the public health nurses

The questionnaire included 11 statements regarding benefits of the working model in the clinical practice of public health nurses. A more detailed description of each statement is included in Table 2. The majority of the public health nurses said that using the working model, which combined the SDQ psychosocial tool and the Internet‐based parent training programme, promoted preventive nursing care (n = 128, 93%) and helped them to empower the families (n = 126, 91%).

**Table 2:** Benefits of the working model in public health nurses' clinical practice. Based on 138 responses to key statements

Statements	n (%)		
The working model…	Agree	Neither agree nor disagree	Disagree
Promotes a preventive framework for behavioural problems	128 (93)	8 (6)	2 (1)
Increases the ability to identify psychosocial risks	133 (96)	4 (3)	1 (1)
Supports the empowerment of families	126 (91)	10 (7)	2 (1)
Identifies families with specific needs	120 (87)	10 (7)	8 (6)
Helps nurses to ask parents about their concerns	123 (89)	12 (9)	3 (2)
Encourages parenting and family skills	124 (90)	9 (7)	5 (4)
Increases early identification, so that social support can be provided	121 (88)	12 (9)	5 (4)
Provides a suitable technology‐assisted model for child health clinics	117 (85)	18 (13)	3 (2)
Increases the nurses' working development and activities	83 (60)	41 (30)	14 (10)
Strengthens evidence‐based public health nursing	84 (61)	45 (33)	9 (7)
Increases utilisation of research	101 (73)	29 (21)	8 (6)

The public health nurses perceived that the working model helped them to identify families with special needs (n = 120, 87%), in areas such as social support, and provided them with a way to ask parents about any family problems (n = 123, 89%). Most of the nurses agreed that the working model was suitable for child health clinics (n = 117, 85%).

Out of 138 public health nurses, 101 (73%) said that the working model increased the use of research, and over half (n = 83, 60%) agreed that it increased the activity and development of their own work and strengthened evidence‐based nursing (n = 84, 61%).

### Using the SDQ psychosocial identification tool in daily practice

The questionnaire also included questions regarding the use of the SDQ as a part of the working model. The majority (n = 133, 96%) of the 138 nurses used the SDQ psychosocial identification tool as a part of their clinical work, and it was mainly used for communication. Out of 138 public health nurses, 105 (76%) used the tool in co‐operation with a doctor and 102 (73%) for discussions with parents. Half of the public health nurses (n = 69, 50%) used the tool to increase their own knowledge about families' situations. Only 17 (12%) used the findings as a part of child's five‐year‐old health check‐up. This means that majority did not use the tool itself when the child was 5 years of age.

### The perceived benefits of the working model for public health nurses

Based on public health nurses' written descriptions in open‐ended questions, the perceived benefits of the working model formed two categories: first, improvements in the competence of public health nurses to deal with mental health and, second, benefits related to the Internet‐based programme. Table 3 describes the qualitative findings related to the perceived benefits of using the working model.

**Table 3:** Perceived benefits of the working model by public health nurses based on 58 responses

Improvements in the competence of nurses to deal with mental health	Benefits related to the Internet‐based parent training programme
Increases discussions about mental health Increases early identification of psychosocial problems Supports nurses' interactions with parents Enhances the nurses' understanding about well‐being and mental health of the child and family Motivates families to seek help for psychosocial problems	Provides easy access to the low‐threshold parent training programme Supports parenting Supports families' daily lives

### ***Improvements in nurses***'*** competence to deal with mental health***

First, public health nurses said that the working model increased their mental health competence and their knowledge about the need to identify risks at an early age. It also improved their ability to discuss concerns with families. One respondent said: ‘It is easier and more useful to talk about challenges when a parent has identified them in their own evaluation of the child’.

Another nurse stated that the working model enabled them to ‘identify and help families when problems have not yet accumulated’.

The nurses also described how the SDQ psychosocial identification tool, when used as a part of the working model, supported their interactions with parents. The public health nurses felt that the systematic use of the tool increased their understanding of the child and family. They also described the SDQ as being easy to use and playing a key role in the programme as it ‘helps with the conversation and speeds up work’ and provides ‘more information about the family’.

### Benefits related to the Internet‐based parent training programme

Second, the nurses described the need for genuine low‐threshold mental health services for families. They also noted the need for those services to tackle the stigma that still exists regarding children's mental health services, despite the fact that interventions are already fairly easy to access. Participants said that a digitally provided parent training programme might be one solution for this, as it would provide easy access without the need for referrals or difficult bureaucracy. A digital programme would also overcome practical barriers, such as travelling, time off work and childcare arrangements.

One nurse said that: ‘The threshold for this model seems to be lower for parents than, for example, seeking help from a developmental and family counselling clinic’. Others said a digital programme provided ‘easy help for families when there is no specific time that they have to be at the appointment’ and that there were ‘no unnecessary referrals, no workload’.

## Discussion

The study showed that with the help of the psychosocial profile based on the SDQ (psychosocial identification tool), public health nurses were able to increase their own knowledge regarding the psychosocial well‐being of the family. This then enabled the nurses to motivate parents to take part in the Internet‐based parent training programme if their child had a high risk of disruptive behaviour. The public health nurses said that the SDQ tool was easy to use. They also felt that the combination of the tool and the families having the possibility to get low‐threshold, easy‐access parent training was beneficial, since they knew that families would get help if they needed it. This encouraged the public health nurses to discuss the psychosocial well‐being of the family. Overall, this working model was suitable for child health clinics, and it could be used in clinical practice.

It is widely known that there are many underlying aspects causing challenges on multiple levels when implementing interventions or working models in clinical practice 28, 29. Therefore, it is important to understand these aspects in order to achieve effective implementation. Moreover, it is essential to include multicomponent strategies for the implementation 30. Understanding process theories could be beneficial to successful implementation. These theories may give guidelines for planning and organising implementation activities. Process theories may also help us to understand how these activities influence the target group 29. To implement the working model as a part of the daily practice of public health nurses, the public health nurses were trained to use the model, were kept up to date on the progress of the implementation (progress report) and were given summaries of the SDQ with clinical outcome recommendations. The core implementation strategies of our wider study are published elsewhere 18.

In this study, the psychosocial identification tool was the SDQ. Previous studies about using the SDQ in primary care produced similar positive findings 31, 32. Borg et al. 31 concluded that the SDQ was a feasible method for screening children's mental health in Finnish primary health care. A Swedish qualitative study found that the SDQ could be used to share information with parents, primary caregivers and preschool teachers 32. The SDQ is short and easy to use and contains both positive and negative behavioural traits. However, one study reported that participants found the SDQ rather burdensome 31, which was not the case in our study. It seems that in our study the psychosocial profile that the public health nurses received helped them in their daily practice. It seems that this type of tool with a psychosocial profile is beneficial for this purpose.

The working model eased the public health nurses into communicating about psychosocial well‐being and problems with both families and doctors. This is a highly important finding because public health nurses have been reported to lack the confidence to confront mental health problems 6, 8. One suggestion that could promote their self‐confidence in dealing with mental health issues is the training and use of a concrete psychosocial identification tool. It seems that because our working model included a psychosocial profile based on the results from a psychosocial identification tool, this provided us with an excellent opportunity to increase the nurses' professional competence and skills with regard to mental health promotion in child health clinics.

Because studies have shown that parent training is the most effective way to treat disruptive childhood behaviour 13, 14, 15, 16, it has been warranted to develop, study and implement these types of interventions and working models. From the prevention point of view, it is important that low‐threshold treatment can be offered as soon as problems have been identified. Furthermore, early identification and early low‐threshold interventions are greatly needed in primary care. In this study, all of the families whose children had a high risk for disruptive behaviour were offered the Internet‐based parent training programme. The knowledge that families will be offered help if they need it provides further motivation for public health nurses to identify the psychosocial needs of the families. However, those families with children who were found to have a high risk for disruptive behaviour and did not want to participate in the parent training programme should be offered another type of psychosocial support. Primary care is in essential role for doing this.

Overall, it is important to increase the mental health competence of public health nurses in their clinical practice. This requires specific tools, instruments and training. Digitalised methods may be one effective way for this. For example, in future, digitalised psychosocial screening tools will be highly valuable. This will ease and quicken the screening of mental health problems.

This survey can be seen as a first step in finding out how public health nurses perceived this working model implemented in their clinical practice. More studies are needed to achieve a deeper understanding of how this may promote their understanding about mental health and early identification. Children's mental health and psychosocial well‐being in families should be seen as the most important area in prevention. For this reason, more studies and models are needed in this area.

### Limitations

This study had several limitations, including the homogeneous group, as all the public health nurses were females and were experienced public health nurses. Our results can, therefore, be generalised to this type of group, but not to other types of participants. The participants' attitudes towards mental health were not measured and it was not possible to assess their attitudes, which is an important aspect when promoting mental health competence. Interviews would be a highly valuable form of further data collection, as they would help us to develop a deeper understanding of the phenomena. The response rate was 50%, and we may only speculate how the remaining half perceived the use of the working model and the psychosocial identification tool. It is possible that the public health nurses' perceptions would not be as positive to those who participated. Based on implementation literature, the entire target group should be committed to it 29. Unfortunately, we only got responses from half of our target group. Therefore, further study on this topic, using qualitative methods, for example, would be prudent. Finally, the instrument that was used to collect data from public health nurses has not been validated. However, the questions were based on the professional competence areas required of Finnish national public health nurses 23, which formed a central element of this study.

## Conclusion

Our findings suggest that public health nurses working in child health clinics were ready, willing and able to use a working model that supported the parenting skills needed to manage the behavioural issues of 4‐year‐old children and ensure their psychosocial well‐being. The working model comprised the SDQ to identify children at risk and the Internet‐based intervention to provide parent training with the added element of weekly telephone sessions with a trained coach. In practice, public health nurses should be systematically trained and supported and continuously motivated so that they develop their ability to deal with mental health problems. This working model enabled our participants to tackle challenges in psychosocial well‐being at a critical transitional period in child development. On the other hand, there is still a gap between clinical practice and systematic use of evidence‐based models for mental health well‐being. Therefore, the crucial question is the following: what kinds of actions are needed to make use of mental health evidence‐based models as systematic as those intended for physical well‐being?

## Conflict of interest

The authors have no conflicts of interest to declare.

## Author contributions

All authors agreed the final version and met at least one of the IJME criteria (http://www.icmje.org/recommendations/). All authors made substantial contributions to the conception and design of the study and the acquisition and interpretation of the data. All the authors drafted the article or critically revised it for intellectual content.

## Ethical considerations

This research was part of a larger implementation study and therefore was covered by the ethical approval (25/2018) from the University of Turku gained for the wider study.
